# Effects of Plant Oil Interesterified Triacylglycerols on Lipemia and Human Health

**DOI:** 10.3390/ijms19010104

**Published:** 2017-12-30

**Authors:** Andreina Alfieri, Esther Imperlini, Ersilia Nigro, Daniela Vitucci, Stefania Orrù, Aurora Daniele, Pasqualina Buono, Annamaria Mancini

**Affiliations:** 1 Dipartimento di Scienze Motorie e del Benessere, Università degli Studi di Napoli “Parthenope”, via Medina 40, 80133 Napoli, Italy; andreina.alfieri@uniparthenope.it (A.A.); orru@uniparthenope.it (S.O.); 2 Ceinge-Biotecnologie Avanzate S.c.a r.l., Via G. Salvatore 486, 80145 Napoli, Italy; nigro@ceinge.unina.it (E.N.); aurora.daniele@unicampania.it (A.D.); 3 IRCCS SDN, via E. Gianturco 113, 80142 Napoli, Italy; esther.imperlini@unina.it (E.I.); vitucci@ceinge.unina.it (D.V.); 4 Dipartimento di Medicina e di Scienze della Salute “Vincenzo Tiberio”, Università degli Studi del Molise, 86100 Campobasso, Italy; 5 Dipartimento di Scienze e Tecnologie Ambientali Biologiche Farmaceutiche, Università della Campania “Luigi Vanvitelli”, Via G. Vivaldi 42, 81100 Caserta, Italy

**Keywords:** fatty acids (FAs), interesterified fats, human health, lipemia, stereospecificity, TAG structure

## Abstract

The position of the fatty acids (*sn*-1, *sn*-2 and *sn*-3) (stereospecific numbering (*sn*)) in triacylglycerol (TAG) molecules produces a characteristic stereospecificity that defines the physical properties of the fats and influences their absorption, metabolism and uptake into tissues. Fat interesterification is a process that implies a positional distribution of fatty acids (FAs) within the TAG molecules, generating new TAG species, without affecting the FA *cis*-*trans* natural balance. The interesterified (IE) fats, frequently used in the food industry comprise fats that are rich in long-chain saturated FAs, such as palmitic acid (16:0) and stearic acid (18:0). Within the interesterified fats, a critical role is played by FA occupying the *sn*-2 position; in fact, the presence of an unsaturated FA in this specific position influences early metabolic processing and postprandial clearance that in turn could induce atherogenesis and thrombogenesis events. Here, we provide an overview on the role of TAG structures and interesterified palmitic and stearic acid-rich fats on fasting and postprandial lipemia, focusing our attention on their physical properties and their effects on human health.

## 1. Introduction

Fatty acids (FAs) constitute the main components of fat: triacylglycerols (TAGs), cholesterol esters and phospholipids. They also play a crucial role in physiological processes, like cell maintenance and replication [1]. FA metabolites, such as prostaglandins, thromboxanes and leukotrienes, act as bioactive lipophilic signaling molecules [2]. Moreover, FAs represent a major source of energy and take part into the absorption of fat-soluble vitamins: A, D, E, and K [1,3].

Most animal and plant-derived food products contain fats that ensure satiety, taste, consistency, and stability. Despite fats often being banned in restricted dietary regimens, FAs are instrumental for a proper growth and development and a minimal intake should be ensured in childhood [4,5,6].

The optimal balance of FA contribution to the diet has been a long-standing matter of debate. In fact, along with the above mentioned beneficial effects played by FAs, it is widely known that no more than 20–25% of energy intake should come from FAs and that a high-fat diet increases the risk for cardiovascular diseases (CVD). In these equations, also artificial *trans* and interesterified (IE) FAs have to be taken into account, both of them being obtained by chemical processing in food industries. *Trans* FAs are the side products of partial hydrogenation, performed on plant oil to transform them into solid or semi-solid fats; on the other side, IE fats now are used as a substitute for *trans* fat in a wide range of commonly consumed foods, comprising spreads, baked goods and confectionary products. At the moment, food industries have no legal requirement to specify the type of FAs (*trans*, IE, etc.) on food labels, making it difficult to approximate how frequently they are consumed. In the US, it is estimated that about 3% of the energy input would come from IE fats if these were the only hard fat replacing partially hydrogenated vegetable oils [7].

In this context, it becomes a matter of priority to study the effects of IE fats used in the food industry on human health, discussing the current understanding of IE structural features, their absorption and digestion properties, their effects on fasting and postprandial lipemia.

## 2. Triacylglycerol (TAG) Structures and Chemical Properties in Plant Oil and Animal Fats

FAs are acidic, monocarboxylic linear chains of different lengths: short-chain FAs (bearing 2–5 carbon atoms (C2–5), medium-chain FAs (C6–12), long-chain FAs (C13–20) [8]. According to the degree of saturation of their carbon chains, they can be grouped into three major categories: saturated FAs (SFAs; no double bonds), monounsaturated FAs (MUFAs; one double bond) and polyunsaturated FAs (PUFAs; two or more double bonds) [9]. 

Up to now, many studies have focused on the role of specific dietary FA classes associated with CVD risk. Although it is widely documented that SFA intake is directly associated with high serum concentrations of low density lipoprotein (LDL) cholesterol [10,11], whereas PUFA intake is correlated with lower concentrations [12], some studies reported that LDL concentration is not the only risk factor for CVD and therefore SFA intake may not be predictive of CVD risk [13,14,15]. In particular, the length of the SFA chains play a crucial role, in that CVD serum biomarkers get worse when SFA chain length, mainly ranging from C12 to C18, is shorter—lauric and myristic acids (C12:0 and C14:0, respectively) are more unfavorable than palmitic acid (C16:0), which in turn is more unfavorable than stearic acid (C18:0) [16]. Nevertheless, palmitic acid is the most abundant SFA naturally occurring in animal fats and vegetable oils, followed by stearic acid. A significant improvement in CVD risk can be achieved by replacing SFAs with unsaturated FAs in diets abundant in vegetable oils, such as oleic and linoleic acids (C18:1 and C18:2, respectively) [17]. 

**Figure 1:**
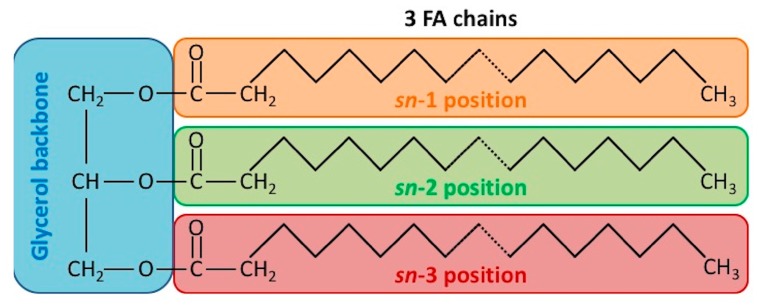
Schematic representation of triacylglycerol molecule structure. Fatty acid (FA); stereospecific numbering (*sn*).

From a chemical point of view, most naturally occurring fats are mixtures of TAGs bearing a glycerol moiety esterified by three FAs, typically palmitic acid, linoleic acid and stearic acid (Figure 1). On the glycerol backbone of TAG molecules, FAs occupy one of three positions, named as *sn*-1, *sn*-2, and *sn*-3, according to the stereospecific numbering (*sn*) system. This stereospecificity determines the physical properties of the TAG, influencing absorption, metabolism and tissue uptake [18]. The occurrence of a centre of asymmetry within TAG structures, allows the existence of enantiomeric forms [19,20,21], whose identification and quantitation require the most advanced analytical technologies, based on tandem mass spectrometry [22,23,24].

Unsaturated FAs, mainly in the *cis* configuration, are the most represented in plant-derived fats that are thus liquid at room temperature, while SFAs are abundant in solid animal fats; unsaturated linoleic acid is, in fact, present in most plant oils, whereas lauric, myristic, and palmitic acids are common in animal fats and only in certain plant oils (palm and coconut). 

To overcome the CVD risk related to SFA intake, food industries took advantage from chemical properties of healthier plant oil TAGs by reducing their double to single bonds through hydrogenation reactions. In fact, such a chemical reduction increases the melting points of oils and allows the transition to solid fats. In the manufacture of soft and semisolid fats, food industries applied a partial hydrogenation, stopping the reaction before all bonds were saturated. But such a practice led to a side effect, namely the isomerization of unsaturated FAs from the native *cis* to the most stable low energy *trans* configuration. On the other hand, *trans* FAs have also been implicated in CVD diseases and the American Heart Association strongly recommends removing them from dietary regimens [7].

The difference between plant oils and animal fats relies also on the overall composition and the stereospecificity of FAs in TAG molecules. The FA composition depends on dietary sources [20]. Oleic acid is the most abundant among the unsaturated FAs, while palmitic acid is the main SFA. Furthermore, the SFA composition may differ between plant and animal fats, even when they both exhibit similar levels of saturation. For instance, cocoa butter contains mainly palmitic and stearic acids, whereas milk fat contains a wider variety of SFAs, including, not only stearic and palmitic acids, but also myristic acid or other medium-chain and short-chain FAs (such as butyric acid) [21]. Moreover, palmitic and stearic acids are present in both beef tallow and palm oil, but in highly different percentages—38% stearic and 50% palmitic acid in beef tallow, whereas 5% stearic and 44% palmitic acids in palm oil [16,19].

The stereospecificity of TAGs is a characteristic hallmark of different natural vegetable oils and animal fats. In these oils and fats, the number of TAG molecules may be theoretically elevated, but actually it is limited by the preferential FA acetylation at specific positions [20]. In fact, the main TAG species in natural fats and oils preferentially retains the same type of FA in the same stereospecific position (Table 1). Generally, most vegetable oils contain SFAs on the *sn*-1 and/or *sn*-3 positions and unsaturated FAs on the *sn*-2 position. Accordingly, in cocoa butter, palm and soybean oils, palmitic and stearic acids are mainly located on the *sn*-1 and *sn*-3 positions, while oleic and linoleic acids occupy the *sn*-2 position [21]. Interestingly, this specific distribution in cocoa butter of native dietary TAGs is responsible for the characteristic melting point of chocolate [25]. Differently, in peanut and olive oils, unsaturated FAs are equally distributed among the three stereospecific positions, while, in coconut oil, the SFAs are widespread in these positions. Unlike vegetable oils, animal fats have SFAs mainly on the *sn*-2 position and unsaturated FAs on the *sn*-1 and/or *sn*-3 positions. In particular, palmitic acid preferentially occupies the *sn*-2 position in bovine milk fat and pork fat (lard), whilst oleic acid is mostly represented at the *sn*-1 and/or *sn*-3 positions (Table 1) [25,26]. Differently, in beef tallow, palmitic acid is mainly located in the *sn*-1 and/or *sn*-3 positions. In horse fat, instead, oleic acid is widespread among the three positions.

## 3. Postprandial Fate of Native and Interesterified Dietary Fats

The stereospecificity of native dietary TAGs, together with FA chain length, influences their digestion and absorption [19]; FAs located in the *sn*-1 and *sn*-3 positions may have different metabolic fates than FAs in the *sn*-2 position. 

The major TAG digestion before absorption occurs in the duodenum by pancreatic lipase, and catalyzes the formation of free FAs (FFAs) and *sn*-2 monoacylglycerols (2-MAGs). Pancreatic lipase hydrolyzes *sn*-1 and *sn*-3 esterified FAs with a higher affinity for the *sn*-1 than the *sn*-3 position [19,20]. Short and medium chain FFAs, originating from the *sn*-1 and *sn*-3 positions, are solubilized in the intestinal environment where they, once adsorbed and complexed with albumin, are transported by portal system to the liver for oxidation [19]. On the other hand, PUFAs located in the *sn*-1 and *sn*-3 positions limit the hydrolytic activity of pancreatic lipase because of their steric hindrance and thus influence the kinetics of FA release [21].

**Figure 2:**
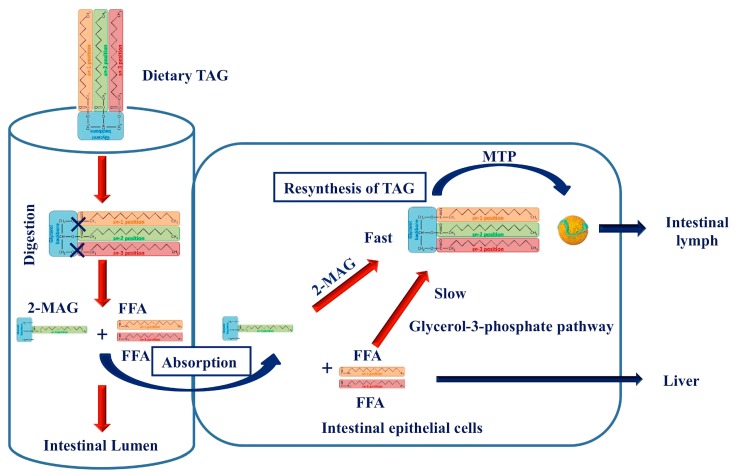
Digestion and absorption of dietary TAG. Dietary TAGs are digested to obtain FFAs and 2-MAGs in intestinal lumen, that are absorbed by intestinal epithelial cells. Here, 2-MAGs rapidly resynthesize TAGs which are included in chylomicron by MTP and transported in the intestinal lymph. FFAs, via glycerol-3-phosphate pathway, are transported by portal system to the liver for oxidation. TAG, Triacylglycerol; 2-MAG, 2-monoacylglycerol; FFA, free fatty acids; MTP, microsomal triglyceride transfer protein.

Absorption of 2-MAGs occurs by passive diffusion into the enterocytes [27], and there they are first recycled for the synthesis of new TAGs and reassembled into lymph chylomicrons at the endoplasmic reticulum level, keeping the native stereospecificity (Figure 2) [18,25,28]. Similarly to pancreatic lipase, lipoprotein lipase (LPL), responsible for highly efficient hydrolysis of TAG-chylomicrons, is also region specific for the *sn*-1 and *sn*-3 positions—the obtained 2-MAG is isomerized to 1(3)-MAG from which FA is efficiently released [29]. Absorbed MAGs can also serve as a primary structure for gut or liver phospholipid synthesis in excessive FFA environments [25].

These findings suggest that TAGs with SFAs in the *sn*-2 position are digested completely, absorbed more efficiently by intestinal epithelium, delaying entry into the blood circulation, compared to TAGs containing SFAs in positions *sn*-1 and *sn*-3 (Figure 2), thus determining a reduced postprandial lipemic response [18,26,30]. 

In addition, FAs may exhibit different absorption patterns, based on chain length and saturation, as well as on their stereospecific position on TAGs. Unsaturated FAs and medium chain FAs are more efficiently absorbed than long chain SFAs. Animal studies showed that fat absorption from vegetable oils containing SFAs on the *sn*-1 and *sn*-3 positions occurs at a lower rate than that of animal fats with SFAs on the *sn*-2 position [31,32]. Long chain SFAs, such as palmitic acid, have low coefficients of absorption because of their melting points above body temperature. Moreover, when palmitic and stearic acids, located at the *sn*-1 and *sn*-3 positions, are released by digestive lipases, they are able to form insoluble calcium or magnesium soaps, due to the alkaline intestinal environment [16,19,21]. This aspect is a relevant issue for infant nutrition, in terms of fat and calcium absorption, whose optimization, as confirmed by food industry and clinical applications, has required the use of dietary TAGs with IE FAs. Interesterification allows modification of the FA position on TAG molecules. In fact, IE FAs are obtained by enzymatic or chemical methods that, at low temperatures, promote the incorporation of a specific FA on the glycerol backbone or the rearrangement of native FAs on the different positions of TAG molecules [33,34,35,36]. This consists of a randomization process in which FAs are nearly equally distributed among the three stereospecific positions of the IE TAG. The interesterification, however, does not alter the unsaturation/saturation degree or the isomeric state of FAs, but only the position of acyl groups on TAG molecules. Although the FA profile is overall unchanged, the physical properties (melting temperature and crystallization characteristics) of IE TAGs are modified and, as a consequence, their absorption properties are also modified. Moreover, the interesterification process can be applied to two or more different native fats (for example, solid fat and liquid oil) giving rise to a completely new TAG mixture. Food industries use interesterification to produce functional ingredients to be incorporated into foods; this also represents a valid alternative to the partial hydrogenation of vegetable oils in the production of margarines, cooking fats and shortenings. In clinical applications, instead, interesterification is used not only to provide products well-absorbed by infants or patients with fat malabsorption disorders, but also to design poorly absorbed fats recommended for weight loss [37]. 

As for infant nutrition, fat absorption from human milk has a higher rate than that from infant formula, partly due to the preferential presence of palmitic acid at the *sn*-2 position of the TAG glycerol backbone [37]. In infants, in fact, the palmitic acid, once absorbed, is esterified to TAG and secreted into plasma. Therefore, the specific *sn*-2 position of palmitic acid in human milk may influence chylomicron formation after digestion and also the metabolism of cholesterol esters and long chain PUFAs [38]. Different from human milk, in conventional infant formula, palmitic acid is mainly located in the *sn*-1 and *sn*-3 positions and absorbed as non-esterified FA. To enhance fat absorption, similarly to human milk, infant formula products contain IE TAGs (such as betapols) with up to 60% or more of palmitic acid at the *sn*-2 position [39,40,41]. Hence, the biological properties of native and IE dietary fats are strictly dependent on the stereospecific position of FAs, namely the presence of SFAs vs. MUFAs/PUFAs at the *sn*-2 position of TAGs influences their digestion and subsequent absorption, and metabolism [18,25].

Likewise, the food industry needed a replacement for hydrogenated FAs, previously developed as a cheaper alternative to animal fats, in an attempt to obtain solid or semi-solid vegetable fats, but also able to impair the naturally-occurring balance between *cis* and *trans* configurations in unsaturated FAs [42]. Successively, the occurrence of a positive association between hydrogenated fat intake and CVD risk induced a progressive reduction of industrial hard vegetable *trans* fats use in the food market, favoring, in parallel, the interesterification process [11,43]. Nowadays, such a process, mainly based on chemical methods, represents a valid option for the food industry to modify the texture and enhance the taste of new dietary products; in general, interesterification is largely used in commonly consumed foods, and this process has allowed the SFA content in respect to non-IE fat to be reduced by 10% [44]; such a promising acknowledgment has pushed even more interest on the health impact of these molecules. 

## 4. Effect of Interesterified Fats on Fasting and Postprandial Lipemia

Several studies have reported the effects on fasting and postprandial lipemia on the positional distribution of palmitic and stearic acids in TAGs modified by interesterification [45]. Generally, fasting is defined as the absence of, or minimum, food intake, for a time period ranging from 12 h to a few weeks; in recent years, fasting regimens have been associated with functional improvements in myocardial infarction, diabetes and stroke. This practice has highlighted the role of fasting in the adaptive cellular response, by means of significant changes in several metabolic and cellular processes, such as stress resistance and inflammation reduction [46]. Regarding the effects of IE FAs on fasting and postprandial lipemia, most reported data refer to a fasting state, defined as no food intake for 6–8 h before blood collection [47,48]. In particular, elevated postprandial lipemia with non-fasting hypertriglyceridaemia is associated with a higher risk of vascular events [49]. This condition may be defined by increased concentrations of circulating TAGs and triyglyceride-rich lipoproteins (TRLs) over time, following the consumption of a meal. High serum concentrations of lipoproteins and TAGs and fasting glucose are all important risk factors for dysmetabolic diseases [50]. Furthermore, impaired blood lipid profiles, together with high serum levels of coagulation activation factors, such as factor VIIa, during the postprandial period, increase the risk of severe atherothrombotic events [30,51].

Early studies reporting the effects of IE fats on fasting lipemia are conflicting. In fact, studies conducted in rats and in rabbits demonstrated that a lipid diet, with different positional distribution of FAs in TAG structure had no effect on the fasting lipid profile [52,53]. Conversely, more recent studies, conducted in both rats and humans, demonstrated that diets containing TAGs bearing SFAs in the *sn*-2 position have a positive influence on plasma lipoprotein profiles and on the reduction of plasma TAG concentrations [25,26,54,55]. Such a reduction can be explained by the different absorption pathway of 2-MAGs bearing SFAs, as shown in Figure 2. 

Furthermore, many studies have compared the effects of a diet rich in IE FAs to a diet rich in native FAs, on lipid profiles, in adult subjects. Native shea butter (stearic predominantly in *sn*-1,3 positions) and IE shea butter were used to study the effects on fasting and postprandial lipemia. All cholesterol fractions (total cholesterol, LDL- and high density lipoprotein (HDL)-cholesterol) and serum TAGs, together with glucose and insulin concentrations, resulted in no modification after the acute administration of two meals with different fat compositions, in a randomized crossover study in adult subjects [56]. Similar results were obtained in other studies, using native and IE butter, native and IE palm oil, or mixtures of natural and IE fats [57,58,59]. 

IE palm oil, with a higher percentage of palmitic acid (70.5%) in the *sn*-2 position, is able to reduce postprandial lipemia in young people with normolipidemic profiles—both male and female subjects [60]. These positive effects are also found in elderly subjects with high fasting TAG concentrations. In this regard, Hall and colleagues [61] compared the effects of high fat (IE palm oil) meal consumption to meals containing native palmate olein, on lipemia and plasma lipoprotein fractions, in men aged 40–70 years with high fasting TAG concentrations. After the first 4 h of the postprandial period, a significant decrease was reported in plasma TAG concentration in response to consumption of IE palm oil vs. native palmate olein. After 4–6 h in the postprandial period, chylomicron concentrations were slightly reduced in subjects consuming IE fat; no differences in plasma intermediate-density-lipoprotein (IDL)-cholesterol fraction or apolipoprotein B48 were observed between the two groups. Thus, consuming a meal containing IE palm oil with a greater proportion (45.9% vs. 9.8%) of palmitic acid in the *sn*-2 position decreases postprandial lipemia, when compared to consumption of a meal containing native palm oil, in the initial postprandial period (4 h) in subjects with higher fasting TAG concentrations [61]. Likewise, the same authors conducted a randomized double-blind crossover study on 12 healthy males (18–45 years), comparing the effects of a single meal, containing a commonly-consumed IE palmitic acid-rich fat mixture (palm stearin and palm kernel), vs. a meal containing non-IE fats. Unexpectedly, in early postprandial period (4 h), plasma TAG concentration was higher in subjects assuming the IE fat-rich meal; after 4 h, this concentration decreased. On the other hand, plasma TAG concentration increased constantly in the group eating native fats, both in early and late postprandial periods. This apparent contradiction could be explained by the fact that the interesterification process affects the content of solid fats and the stereospecific position of FAs in TAGs [62]. In agreement with these results, a recent study performed on young healthy subjects demonstrated that the consumption of high fat meals, rich in TAGs, containing palmitic acid at the *sn*-2 position, decreased postprandial lipemia, when compared to the consumption of high oleic sunflower oil meals [60]. Furthermore, gender differences were observed in plasma TAG concentrations, in response to the test meals; in fact, the postprandial increase in plasma TAGs was, in general, lower in women compared to age-matched men [40]. Successively, in a randomized crossover study on 17 healthy men, the same authors reported a reduction in postprandial lipemia after consumption of IE cocoa butter, compared with the consumption of native cocoa butter [63]. Lastly, Berry et al. [64] compared the effects of IE palm oil vs. native palm oil and IE palm oil vs. high-oleic sunflower oil consumption in two randomized clinical trials conducted on healthy men subjects, on the plasma lipid profile, glucose, insulin and VIIa factor concentrations, respectively. The authors demonstrated that IE palm oil consumption decreased postprandial plasma TAG and insulin concentrations, when compared both with native palm oil and sunflower oil consumption; IE palm oil consumption also reduced of 64% the postprandial VIIa factor plasma concentration at 6 h. No significant differences in plasma insulin concentrations or in chylomicron composition were observed in any trial [64]. 

## 5. Conclusions

IE fats have largely replaced *trans* fats in processed foods and the interest on their effects concerning the metabolic fate and the potential impact on human health is rapidly growing. This review provides an overview on the effects of IE fats, currently used in human nutrition, on fasting and postprandial lipemia, and highlights that further studies are needed in order to clarify their impact on human health. In fact, few data regarding the effects of randomized FAs on fasting plasma lipemia are available so far; however, they indicate that the consumption of meals with IE fats does not influence, or is able to reduce, fasting lipemic profiles compared to consumption of meals containing a mixture of native fats with the same FA profile. Such a finding, if confirmed in future studies, could represent a favourable feature in the consumption of IE fats that does not concur with CVD risk. In addition, data on the effects of randomized FAs on postprandial lipemia are in agreement with reporting a reduction of postprandial plasma TAG concentrations, in young, adult and elderly subjects, with a more pronounced effect in women compared to age-matched men. Hence, also in postprandial conditions, IE fat-rich meals could be able to reduce CVD risk [65,66,67]. Nevertheless, as quoted above, plasma TAG concentrations and cholesterol fractions are not the only measurable factors related to CVD risk, and so other independent causes have to be evaluated [13,14,15].

Overall, since clinical studies on IE fats have, in general, been performed using levels of fat intake exceeding the normal daily consumption and using commercially unavailable products [44], we highly support the need to build standardized nutrition trials, in order to explore the acute and chronic potential effects of IE fats, not only on CVD, but also on inflammation and cancer.

## Figures and Tables

**Table 1:** Distribution of FAs in TAG molecules of chosen plant oils and animal fats.

Plant Oil	TAG			Animal Fat	TAG		
Type	*sn*-1	*sn*-2	*sn*-3	Type	*sn*-1	*sn*-2	*sn*-3
Cocoa butter	P	O	P	Milk (cow)	P	P	B
	P	O	S		O	P	B
	S	O	S		P	M	B
Palm oil	P	O	P	Lard (pig)	S	P	O
	P	O	L		O	P	L
	P	O	O		O	P	O
Soybean oil	L	L	P	Tallow (beef)	P	O	P
	L	L	L		P	S	O
	L	L	O		P	O	O
Peanut oil	P	O	L	Butter	P	P	B
	O	L	L		P	P	C
	O	O	L		P	O	P
Olive oil	O	O	P	Horse fat	P	O	O
	O	O	O		O	O	O
	O	L	O		L	O	O
Coconut oil	D	D	D	Egg	P	O	O
	C	D	D		P	L	O
	C	D	M		P	O	S

Abbreviations used for fatty acids (FAs) on the three position of triacylglycerols (TAGs): P, palmitic acid; O, oleic acid; S, stearic acid; L, linoleic acid; D, dodecanoic acid; M, myristic acid; B, butyric acid; C, capric acid.
